# Rac-GTPases Regulate Microtubule Stability and Axon Growth of Cortical GABAergic Interneurons

**DOI:** 10.1093/cercor/bhu037

**Published:** 2014-03-13

**Authors:** Simona Tivodar, Katerina Kalemaki, Zouzana Kounoupa, Marina Vidaki, Kostas Theodorakis, Myrto Denaxa, Nicoletta Kessaris, Ivan de Curtis, Vassilis Pachnis, Domna Karagogeos

**Affiliations:** 1 Institute of Molecular Biology and Biotechnology (IMBB, FORTH), Heraklion, Greece; 2 Department of Basic Science, Faculty of Medicine, University of Crete, Heraklion, Greece; 3 Division of Molecular Neurobiology, Medical Research Council, National Institute for Medical Research, London, UK; 4 Wolfson Institute for Biomedical Research and Department of Cell and Developmental Biology, University College London, UK; 5 Cell Adhesion Unit, Dibit, San Raffaele Scientific Institute, 20132 Milano, Italy; 6 Current Address: Koch Institute for Integrative Cancer Research at MIT, Massachusetts Institute of Technology, Cambridge, MA 02139, USA

**Keywords:** cortical development, cytoskeleton, medial ganglionic eminence, Rho-GTPases

## Abstract

Cortical interneurons are characterized by extraordinary functional and morphological diversity. Although tremendous progress has been made in uncovering molecular and cellular mechanisms implicated in interneuron generation and function, several questions still remain open. Rho-GTPases have been implicated as intracellular mediators of numerous developmental processes such as cytoskeleton organization, vesicle trafficking, transcription, cell cycle progression, and apoptosis. Specifically in cortical interneurons, we have recently shown a cell-autonomous and stage-specific requirement for Rac1 activity within proliferating interneuron precursors. Conditional ablation of Rac1 in the medial ganglionic eminence leads to a 50% reduction of GABAergic interneurons in the postnatal cortex. Here we examine the additional role of Rac3 by analyzing Rac1/Rac3 double-mutant mice. We show that in the absence of both Rac proteins, the embryonic migration of medial ganglionic eminence-derived interneurons is further impaired. Postnatally, double-mutant mice display a dramatic loss of cortical interneurons. In addition, Rac1/Rac3-deficient interneurons show gross cytoskeletal defects in vitro, with the length of their leading processes significantly reduced and a clear multipolar morphology. We propose that in the absence of Rac1/Rac3, cortical interneurons fail to migrate tangentially towards the pallium due to defects in actin and microtubule cytoskeletal dynamics.

## Introduction

γ-Aminobutyric acid-producing (GABAergic) interneurons provide the main source of inhibition to cortical circuits and their impaired function underlies severe neurodevelopmental disorders such as schizophrenia, epilepsy, and autism (Le Magueresse and Monyer 2013). Cortical interneurons originate in the ganglionic eminences, well-defined domains of the subpallial ventricular zone (VZ), from where they migrate tangentially to populate the different layers of the neocortex. Cortical GABAergic interneurons can be divided into different subpopulations according to distinct morphological, molecular, and functional properties. The most recently proposed classification divides interneurons in 3 nonoverlapping groups defined by the expression of parvalbumin (PV), somatostatin (Sst), and the ionotropic serotonin receptor 5HT3a (5HT3aR) (Rudy et al. 2011).

The medial ganglionic eminence (MGE) is the major source of PV^+^ and Sst^+^ cortical GABAergic interneurons (Fishell and Rudy 2011). Nkx2.1, a homeobox transcription factor, is a key regulator for the specification of interneuron populations in the MGE (Sussel et al. 1999; Xu et al. 2004; Butt et al. 2008). Downstream of Nkx2.1, Lhx6, a LIM homeodomain protein, is required for the tangential migration of GABAergic interneurons in the cortex and the specification of PV and Sst interneurons (Liodis et al. 2007).

Extracellular factors determine the interneuron migratory routes by modifying their leading processes through the activation of intracellular pathways. New leading process branches are generated in response to chemoattractant cues, in order to change the direction of migration (Martini et al. 2009). Rac proteins, a subfamily of Rho-GTPases, are crucial players in processes such as cytoskeleton organization, vesicle trafficking, transcription, cell cycle progression, and apoptosis (Jaffe and Hall 2005; Gonzalez-Billault et al. 2012).

Most of our knowledge on Rac protein function is based on work focused on the ubiquitously expressed Rac1. Particularly in the central nervous system, conditional knock-out of Rac1 in the VZ of the telencephalon results in axonogenesis defects and impaired migration of cortical cells (Chen et al. 2007, 2009). Rac1 is known to regulate neuronal polarization, migration, and axon growth via the WAVE complex in cerebellar granule neurons that express only one member of the Rho-GTPase family, Rac1 (Tahirovic et al. 2010). In addition, in dissociated cultures of hippocampal pyramidal cells, Rac1 activity occurs downstream of the microtubule-associated protein MAP1B (Montenegro-Venegas et al. 2010). We have recently demonstrated that Rac1 is required cell autonomously for cortical interneuron development (Vidaki et al. 2012). Mice lacking Rac1 exclusively in MGE-derived cells exhibit a 50% reduction in the number of GABAergic interneurons found in the postnatal cortex. Rac1 is required in cycling progenitors, since in its absence they are significantly delayed in exiting the cell cycle, probably due to a longer G1 phase. Mutant GABAergic interneurons also show abnormal axon growth cone morphology in vitro. These data demonstrate that Rac proteins are important players in interneuron development through diverse functions (Vidaki et al. 2012).

In contrary to the information about Rac1 function in diverse cell types, little is known about Rac3, which is highly expressed in the nervous system (Malosio et al. 1997). Mice deficient for Rac3 do not show impaired cortical development and new-born mice do not show any obvious phenotypes, while the results of behavioral tests on motor coordination and learning showed some difference between control and Rac3-deficient mice (Corbetta et al. 2005). The ablation of Rac1 and Rac3 from differentiated neurons leads to the loss of mossy cells and to specific alterations in vivo and in vitro such as dendritic spine formation of hippocampal neurons as well as a reduction of cortical and hippocampal interneurons (Corbetta et al. 2009; Vaghi et al. 2012).

We asked whether Rac3 may compensate for the lack of Rac1 in early interneuron development, since half of them still manage to populate the mature cortex in Rac1 mutants. In this report we validate this hypothesis and analyze the molecular basis of the observed phenotypes. We have bred the Rac3^−/−^ (Corbetta et al. 2005) mice to the Rac1^fl/fl^;Nkx2.1^+/Cre^ line (Vidaki et al. 2012) which deletes Rac1 from MGE progenitors, to ask whether the absence of neuronal specific Rac3 in the Rac1-deficient interneurons has additive and/or distinct effects. We observed that the reduction of GABAergic interneurons is severely more pronounced in the double mutant than in the Rac1 mutant alone. Interestingly, our in vitro and in vivo analyses suggest that the further decrease in the number of GABAergic interneurons in the postnatal cortex of double Rac1/Rac3 mutants is due to migratory defects. In particular, we show that the leading processes of Rac1/Rac3-deficient MGE-derived cells have distinct cytoskeletal deficits. We correlate these defects with a reduction in the amount of the stable fraction of microtubules. Our data are consistent with the hypothesis that Rac1 together with Rac3 play a role in stabilizing microtubules, thus influencing axonal growth/polarity of MGE-derived interneurons. Furthermore, these findings indicate that Rac proteins connect and coordinate crucial events for cortical interneuron development such as the organization of actin and microtubule cytoskeleton.

## Materials and Methods

### Mice

Animals carrying a floxed allele of Rac1 (Rac1^fl/fl^;Nkx2.1^+/Cre^) were previously described (Vidaki et al. 2012). To obtain double-mutant animals for Rac1 and Rac3, the Rac1 ^+/fl^;Nkx2.1^+/Cre^ line was crossed with the Rac3 ^KO^ line (Corbetta et al. 2005). The ROSA26^fl-STOP-fl-YFP^ allele was also inserted as an independent marker, to allow visualization of the Rac1/Rac3 mutant (and control) neurons, via yellow fluorescent protein (YFP) expression (Srinivas et al. 2001). Rac1^fl/fl^;Rac3^−/−^;Nkx2.1^Tg(Cre)^; R26R-YFP^+/−^ and Rac1^+/fl^;Rac3^+/−^;Nkx2.1^Tg(Cre)^;R26R-YFP^+/−^ animals will be referred to as Rac1^fl/fl^;Rac3^−/−^;Nkx2.1^+/Cre^ (double mutant; dmut) and Rac1^+/fl^;Rac3^+/−^;Nkx2.1^+/Cre^ (control; dhet) respectively, in Materials and Methods, text, figures, and legends. Dhet and wild-type animals (Rac1^+/+^;Rac3^+/+^;Nkx2.1^Tg(Cre)^;R26R-YFP^+/−^) were indistinguishable in all tests performed (data not shown). In parallel, the comparison of dhet was made with Rac1^+/fl^;Rac3^−/−^;Nkx2.1^Tg(Cre)^;R26R-YFP^+/−^ (named Rac3 mutant, Rac3mut) animals to assess the involvement of Rac3 alone and (Rac1^fl/fl^;Rac3^+/−^;Nkx2.1^+/Cre^, named Rac1 mutant, Rac1mut) whenever comparisons with conditional Rac1 mutant animals were necessary.

The genotyping was performed by PCR, using specific primers for:

Rac3

1. CATTTCTGTGGCGTCGCCAAC

2. CACGCGGCCGAGCTGTGGTG

3. TTGCTGGTGTCCAGACCAAT

For timed pregnancies, the day of the vaginal plug was designated as embryonic day E0.5 and the day of birth was considered as P0.

Most double-mutant mice die 1–2 weeks earlier than the Rac1 conditional mutants, at postnatal day 5 (P5). In some cases they survive until 2 weeks. At birth, the double mutants are smaller than the control pups and this difference is maintained until P15 which is the last age examined as the animals die after P15. Mice are able to feed themselves as milk is evident in their stomachs but are observed to have epileptic-like seizures which we are currently investigating; our working hypothesis is that epilepsy is the underlying cause of death. The colony is maintained in the animal facility of the Institute of Molecular Biology and Biotechnology (IMBB-FORTH), Heraklion, Crete, Greece. All experiments were approved by the General Directorate of Veterinary Services, Region of Crete, Greece.

### Reverse Transcription-PCR

Total RNA extraction from the MGE of E13.5 wild-type (+/+) and Rac3 knockout (−/−) embryos was performed with Trizol (Invitrogen), and followed by cDNA reverse transcription using the Affinity Script Multiple Temperature cDNA Synthesis Kit (Agilent), according to the manufacturer's instructions. cDNA amplification has been performed using the Phusion High-Fidelity DNA Polymerase (FINNZYMES) and specific primers for Rac3 (forward, 5′-CCGCTCGAGATGCAGGCCATCAAGTG-3′ and reverse, 5′-CTAGCTAGCCTAGAATACAGTGCTCTT-3′) or GAPDH (forward, 5′-ATTGTCAGCAATGCATCCTG-3′ and reverse, 5′-ATGGACTGTGGTCATGAGCC-3′). The following amplification protocol was used: 98°C for 30 s and 32 cycles of 98°C for 7 s, 57°C for 20 s, and 72°C for 20 s and final extension for 5 min at 72°C. Products were electrophoresed on a 2% agarose gel.

### Immunohistochemistry

Embryonic brains at different ages from E12.5 and onwards, and postnatal P5 brains were dissected in PBS and fixed in 4% paraformaldehyde (PFA) overnight. P15 pups were perfused with 4% PFA/0.25% glutaraldehyde following fixation with the same solution for 1 h at 4°C. They were subsequently processed as previously described (Vidaki et al. 2012).

Primary antibodies used: rabbit polyclonal anti-GFP (Minotech biotechnology, Heraklion, Greece, 1:5000), rat monoclonal anti-GFP (Nacalai Tesque, Kyoto, Japan, 1:500), rabbit polyclonal anti-Lhx6 ((Liodis et al. 2007) 1:200), rabbit polyclonal anti-GABA (Sigma, Saint Louis, MI, 1:1000), rabbit polyclonal anti-PV (Swant, Bellinzona, Switzerland; 1:1000), rabbit polyclonal anti-calretinin (CR) (Swant, Bellinzona, Switzerland, 1:1000), rat monoclonal anti-Sst (Abcam, Cambridge, MA, 1:500), rat polyclonal anti-BrdU (Oxford Biotech, Oxford, UK, 1:1000), rabbit polyclonal anti-Ki67 (Vector Laboratories, Burlingame, CA, 1:1000), rabbit anti-cleaved Caspase 3 (Cell signaling, Beverly, MA, 1:200), Phalloidin-Alexa 595 (Invitrogen), rabbit anti-GAPDH (Cell Signaling, 1:1000), mouse anti Ac-Tubulin (Sigma, Saint Louis, MI, 1:200), rat anti Ty-Tubulin (Abcam, Cambridge, UK, 1:50), mouse anti-Tau1 (Millipore, Billerica, MA, 1:400). Secondary antibodies used: goat anti-mouse-Alexa Fluor-488, -555, or -633, goat anti-rabbit-Alexa Fluor-488, -555, or -633 and goat anti-rat-Alexa Fluor-488, -555, or -633 (all from Molecular Probes, Eugene, OR, all 1:800).

### BrdU Incorporation and Staining

Pregnant females of designated embryonic stages were injected intraperitoneally with 50 μg/animal gr, and sacrificed at appropriate stages to collect the embryos. The brains were processed for immunohistochemistry with Ki67 antibody and then they were treated with 2 N HCl to expose the BrdU antigen, as reported in Vidaki et al. (2012).

### Western Blot

MGE and lateral ganglionic eminence from E13.5 forebrain tissue were isolated and lysed as described in Vidaki et al. (2012). Lysates were run on SDS-PAGE and transferred on Nitrocellulose membranes (Whatman GmbH, Dassel, Germany). Membranes were subsequently blocked with 5% BSA in PBS, 0.1% Tween-20 and immunoblotted with mouse anti-AcTubulin (Sigma, 1:5000), and rabbit anti-GAPDH (1:1000) diluted in blocking solution. Secondary antibodies used: anti-mouse-IgG-Horseradish Peroxidase (GE Healthcare, Buckinghamshire, UK, 1:4000).

### RNA In Situ Hybridization

Nonradioactive in situ hybridization on fixed cryostat sections was performed as described previously (Vidaki et al. 2012). Riboprobes used were specific for GAD67, NPY, and Cux2 (kindly provided by Dr F. Guillemot, National Institute for Medical Research, Medical Research Council, Mill Hill), Lhx6, *Sst* (Denaxa et al. 2012), Rac3 (Corbetta et al. 2005), RORβ, and ER81 (kindly provided by Dr M. Studer iBV—Institut de Biologie Valrose, Institut de Biologie Valrose, Nice).

### MGE Matrigel Explants and Dissociated Cell Culture MGE

MGE explants and dissociated cell cultures were prepared from E13.5 embryonic forebrains as previously described (Vidaki et al. 2012).

### Taxol Treatment

MGE-derived cells were plated and after 24 h in vitro the medium was changed with medium containing 50 nM–1 µM taxol for another 48 h in culture.

### SEM

For scanning electron microscopy, MGE-derived cells, after 2 days in culture, were fixed in 2% glutaraldehyde, 2% PFA in 0.08 M sodium cacodylate buffer, pH 7.4, for 24 h at 4°C, washed in the above mentioned buffer, postfixed in 2% aqueous OsO_4_ for 60 min at 4°C, and dehydrated through a graded series of ethanol. Dehydrated samples were critical point dried (Baltec CPD 030) and mounted on copper stubs prior to sputter coating with 20 nm thick gold/palladium (Baltec SCD 050). Samples were examined using a JEOL JSM-6390LV scanning electron microscope, operating at 20 kV.

### Quantification and Statistical Analysis

The quantification of different interneuron subpopulations in P5 and P15 brains and the cell cycle exit on embryonic sections was previously described (Vidaki et al. 2012).

The Image J program was used for the measurements of the leading process length as well as the angle between the leading process and migration axis in vivo. Twenty cells were randomly picked on each of 3 consecutive sections per animal and the statistical analysis was performed using Student's *t-*test.

The migration distance of the cells out of matrigel explants was measured using arbitrary units in Image J. For each genotype we used at least 3 animals and from each animal 3–4 explants were monitored. All data are presented as mean ± SEM.

In order to quantify the defect on MGE cell cultures when Rac1/Rac3 are absent or only Rac1 is absent, E13.5 littermate embryos were used (Rac1^+/fl^;Rac3^+/−^;Nkx2.1^+/Cre^ dhet, Rac1^fl/fl^; Rac3^+/−^;Nkx2.1^+/Cre^ Rac1 mutant and Rac1^fl/fl^;Rac3^−/−^;Nkx2.1^+/Cre^ dmut) from 4 experiments. The Image J program was used for the measurements of the leading process length on 50–100 cells that were randomly picked. For each genotype the number of leading processes was counted and the data represent mean ± SEM.

The quantification of the intensity of the bands from western blots was made using Image J. The values included in the graph represent the average from 3 experiments after the normalization; in each case, we used pools of material from 4 animals per genotype and the data represent mean ± SEM.

## Results

### Embryonic Migration of MGE-derived Interneurons is Affected in the Absence of Rac1 and Rac3 Proteins

Recently we carried out a microarray-based comparative profiling of gene expression of dorsal forebrain from embryonic day (E) 15.5 wild-type and Lhx6 mutant mice to identify novel genes implicated in cortical interneuron development (Denaxa et al. 2012). Among the genes affected by the deletion of Lhx6 in the dorsal forebrain was the Rho-GTPase protein Rac3. Since Lhx6 null mice show defects in the tangential migration of cortical interneurons (Liodis et al. 2007; Zhao et al. 2008), we hypothesized that this might be partially due to the lack of Rac3 protein in Lhx6 mutant interneurons. Nevertheless, Rac3 mutant mice have been reported not to have obvious defects in cortical interneuron migration (Corbetta et al. 2005), while Rac1-deficient mice show a 50% reduction of GABAergic neurons in the postnatal cortex (Vidaki et al. 2012). This difference may be due to the fact that both Rac1 and Rac3 proteins can compensate for each other's function during interneuron migration. Therefore, we set out to study the role of both Rho-GTPase proteins in cortical interneuron development.

Our first objective was to examine the expression of Rac3 in the forebrain early in development by in situ hybridization experiments. As shown in Supplementary Figure 1, at E13.5, a signal for Rac3 in the wild-type MGE is revealed (Supplementary Fig. 1*A*) while it is totally absent from Rac3 mutant embryos (Supplementary Fig. 1*B*). Independently, Rac3 is also detected by RT-PCR on MGE tissue at this stage (Supplementary Fig. 1*E*) confirming its expression in this area. By E16.5 Rac3 is expressed throughout the developing cortex, mostly confined to the cortical plate (Supplementary Fig. 1*C*), while in the P5 cortex its expression is found in all layers but more intensely in layers IV/V (Supplementary Fig. 1*D* and Fig. 4*B*,*B′*;*G*,*G′*).

In order to study the consequences of the absence of both Rac1 and Rac3 proteins on cortical interneuron development, the Rac1 conditional knockout (Rac1^fl/fl^; Nkx2.1^+/Cre^, [Vidaki et al. 2012]) was crossed to Rac3-deficient mice (Rac3^−/−^, Rac3 KO; [Corbetta et al. 2005]). Thus, we obtained a line where cortical interneurons deriving from the MGE are missing both Rac1 and Rac3 proteins (Rac1^fl/fl^; Rac3^−/−^;Nkx2.1^+/Cre^, double mutant, see Materials and Methods) and the MGE-derived cells expressing Cre are visualized by the Rosa26-YFP reporter.

**Figure 1.**
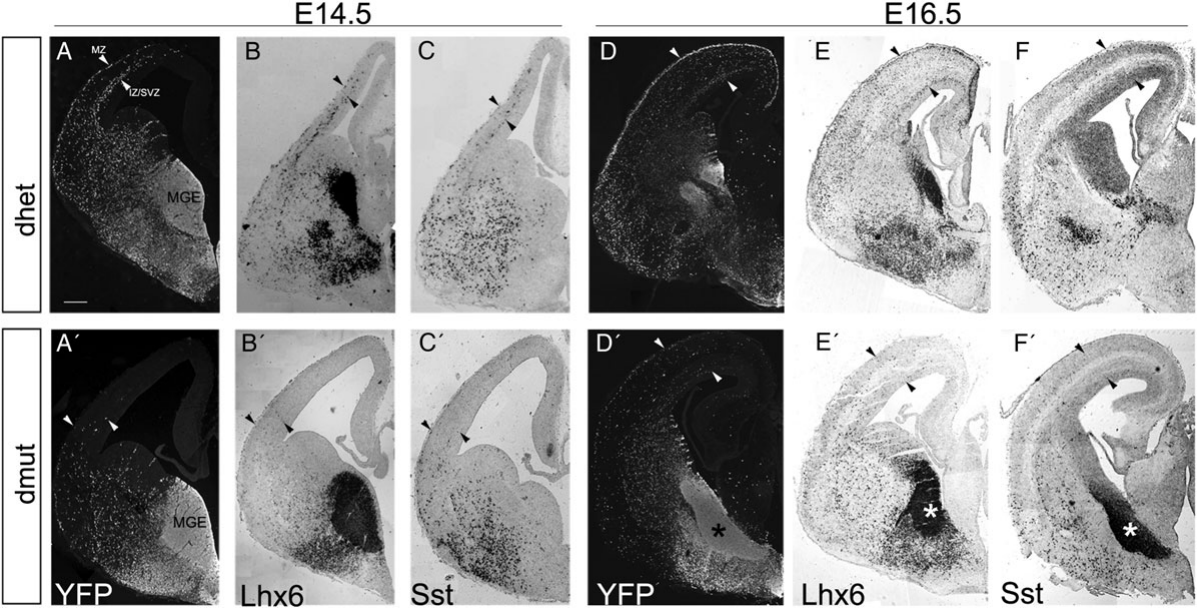
MGE-derived interneurons fail to migrate towards the neocortex in double Rac1/Rac3 mutant embryos. Coronal sections from the forebrain of control and double-mutant embryos at E14.5 (*A–C′*) and E16.5 (*D–F′*), were stained for YFP and specific markers of interneurons to visualize the migration of MGE-derived cells to the developing neocortex. In the double mutants at both ages (*A′–C′*, *D′–F′*: Rac1^fl/fl^;Rac3^−/−^;Nkx2.1^+/Cre^, dmut) Rac1/Rac3-deficient interneurons fail to migrate towards the developing neocortex, compared with the control ones (*A–C*, *D–F*: Rac1^+/fl^;Rac3^+/−^;Nkx2.1^+/Cre^, dhet). At E16.5 some of the Rac1/Rac3-deficient cells migrate towards the cortex although the majority remain aggregated in the ventral telencephalon (asterisk in *D′–F′*) compared with control ones as revealed by immunostaining for YFP and interneuronal markers (Lhx6, *E*, *E′*; Sst, *F*, *F′*). The number of Lhx6 and Sst-expressing interneurons is severely reduced in the cortex of dmut embryos (*B′*, *C′*, *E′*, *F′*) compared with control ones (*B*, *C*, *E*, *F*) at both embryonic stages. Arrowheads indicate the two migratory streams in the MZ and the IZ/SVZ. Scale bars: 100 μm.

Using immunofluorescence on E14.5 forebrain cryosections from control (dhet) embryos we observed YFP^+^ interneurons migrating tangentially forming the characteristic two cellular streams in the marginal zone (MZ) and the intermediate/subventricular zone (IZ/SVZ) and entering the dorsal telencephalon ([Tanaka and Nakajima 2012; Marin 2013]; Figure 1*A,* arrowheads indicate the MZ and IZ/SVZ). These two cellular streams of migrating interneurons were completely absent in the double-mutant embryos (Fig. 1*A′*). Upon comparison of the YFP-stained sections from the double mutant with equivalent sections from the conditional Rac1 mutant (Fig. 1 of (Vidaki et al. 2012) and data not shown) at subsequent stages, we observed that the double-mutant cells entered the dorsal telencephalon ∼1 day later (E14.5, Fig. 1*A′*) than the single Rac1 mutant YFP^+^ cells (Fig. 1 of Vidaki et al. 2012). Analysis with a postmitotic marker of MGE-derived GABAergic interneurons, Lhx6, and a subtype specific marker, Sst verified the severely reduced number of migrating interneurons at E14.5 in the dorsal telencephalon of the double mutants (Fig. 1*B,B′;C,C′*)*.* Later in embryogenesis (E16.5), in the double mutants, only a few YFP^+^, Lhx6^+^, or Sst^+^ cells were found inside the cortex but not extending as dorsally as in the control mice (Fig. 1*D*,*E,F,D′,E′,F′*). In addition, a significant accumulation of these cells was observed in the ventral telencephalon of double-mutant embryos and not in the control ones (asterisk in Fig. 1*D′,E′,F′*).

**Figure 2.**
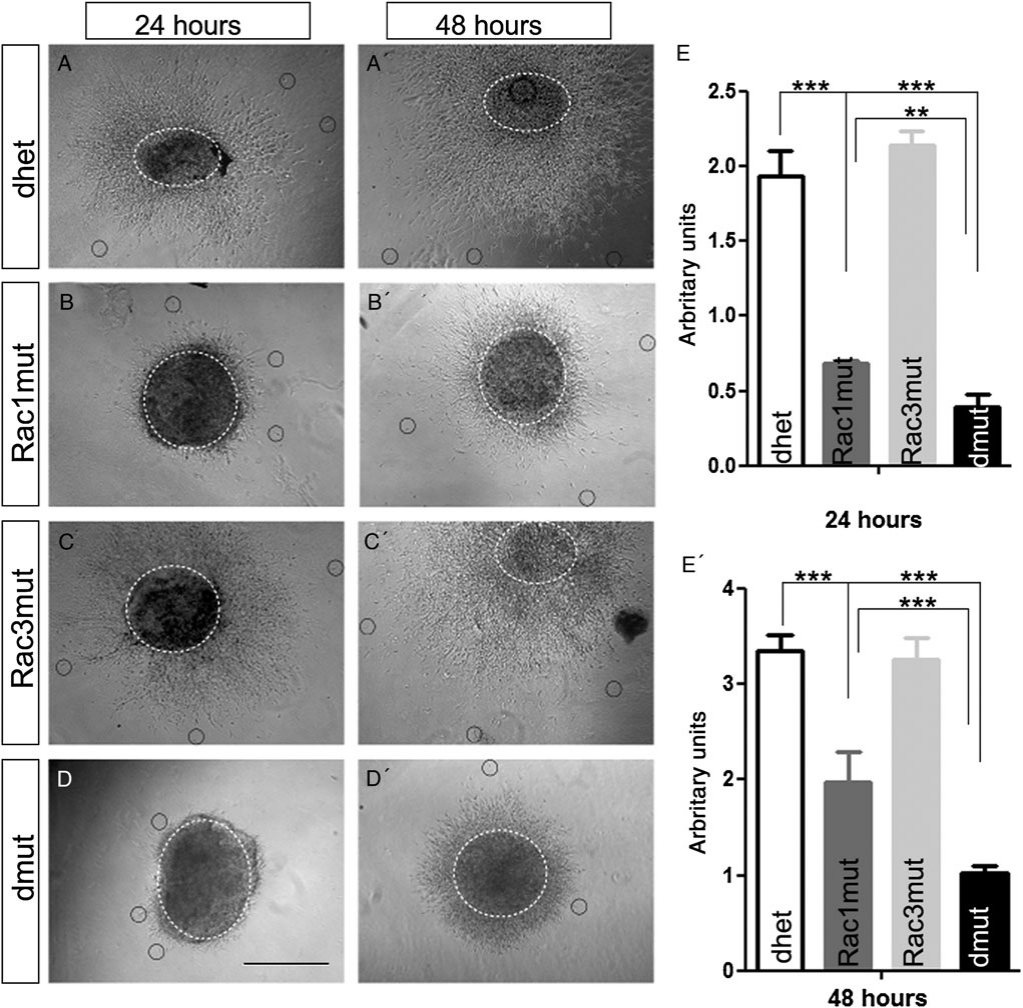
Cell autonomous migration defect of MGE-derived Rac1 and Rac1/Rac3-deficient interneurons. Matrigel explants from E13.5 control (*A*, *A′*: Rac1^+/fl^;Rac3^+/−^;Nkx2.1^+/Cre^, dhet), Rac1 mutant (*B*, *B′*: Rac1^fl/fl^;Rac3^+/−^;Nkx2.1^+/Cre^, Rac1mut), Rac3 mutant (*C*, *C′*: Rac1^+/fl^;Rac3^−/−^;Nkx2.1^+/Cre^, Rac3mut) and double mutant (*D*, *D′*: Rac1^fl/fl^;Rac3^−/−^;Nkx2.1^+/Cre^, dmut) embryos were included in matrigel and cell migration was monitored. After 24 h very few cells were migrating away from the Rac1 mutant and Rac1/Rac3 mutant explants (*B*, *D*) compared with the control or Rac3 mutant explants (*A*, *C*). Reduced migration was observed after 48 h in Rac1 mutant and double-mutant cultures (*B′*, *D′*). The distance of the neurons positioned furthest away from the explants was measured, and statistical significance was assessed, using Student's *t-*test (*P* value ≪0.05; *n* = 10). Error bars represent the standard error of mean (*E*, *E′*). Scale bars: 50 μm.

To further study the migrating behavior of double-mutant cells and investigate whether the observed defects are cell autonomous, we cultured MGE explants on matrigel and analyzed the migration of cells out of the explants. MGE explants were collected from 4 different genotypes: control, Rac1 mutant, Rac3 mutant, and double mutant (see Materials and Methods section describing mice). After 24 h, explants from control or Rac3 mutant (Fig. 2*A,C*) looked similar, with cells migrating from the explant and forming a characteristic halo. Rac1 mutant cells were delayed in leaving the explants (Fig. 2*B*) as described in Vidaki et al. (2012). In double-mutant explants, even fewer cells were migrating for a short distance from the explant compared with the Rac1 mutant (Fig. 2*D,E*) at 24 h. At 48 h, while the control and Rac3 mutant cells were covering comparable areas (Fig. 2*A′,C′*) the distance traveled by the Rac1 mutant and the double-mutant cells (Fig. 2*B′,D′*) was significantly shorter, with the double-mutant cells covering the least distance of all genotypes (Fig. 2*E′*). The differences in distance traveled of double-mutant cells compared with control and Rac1 mutant alone is statistically significant (Fig. 2*E*,*E′*). We observed in our preliminary live imaging experiments that the emerging cells from the double-mutant MGE explants were less motile and did not display the characteristic growth cone extension/retraction movements (data not shown). Thus we consider that the delay in migration may be related to morphological and cytoskeletal abnormalities.

### The Number of MGE-derived GABAergic Interneuron Subpopulations is Severely Reduced in the Rac1/Rac3 Mutant Cortex While Their Differentiation is Unaffected

**Figure 3.**
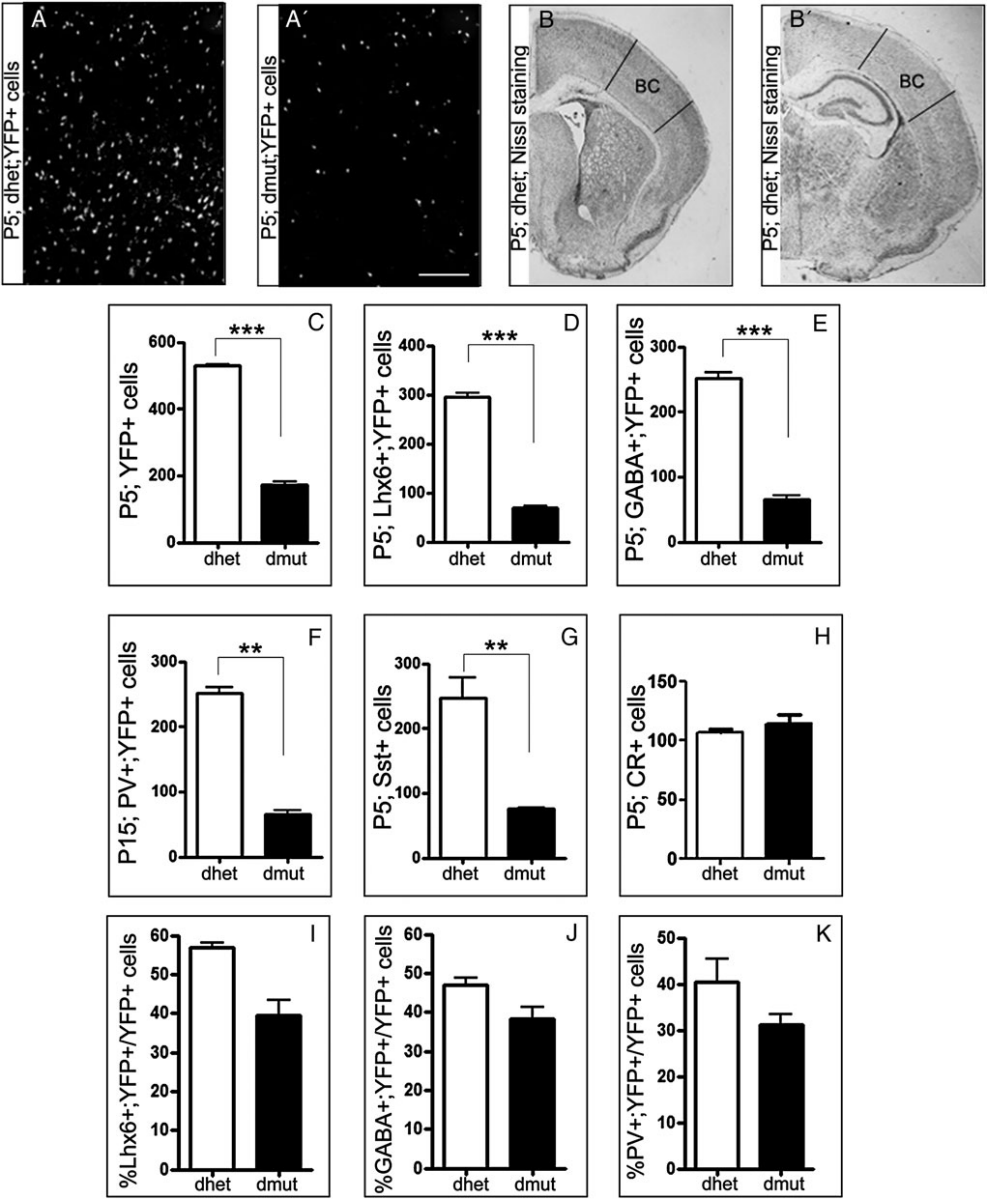
The number of different MGE-derived cortical interneuron subtypes in the Rac1 and Rac3 mutant postnatal barrel cortex is severely reduced. Distribution of different interneuron subtypes in P5 and P15 brains was analyzed using specific markers for GABAergic interneurons. The distribution (*A*, *A′*) and number of YFP^+^ (*C*), Lhx6^+^;YFP^+^ (*D*), GABA^+^;YFP^+^ (*E*), PVA^+^;YFP^+^ (*F*) and Sst^+^ (*G*) interneurons was reduced to almost 80% in the double mutants compared with the control animals. The number of CR^+^ cells was not affected in the absence of Rac1/Rac3 proteins (*H*). The percentage of double positive Lhx6;YFP (*I*), GABA;YFP (*J*) and PVA;YFP (*K*) over the total number of YFP^+^ cells is not different between control and mutant in the barrel cortex. Coronal sections from P5 brains with Nissl staining were taken within the range of bregmata from 0.86 to −1.46 mm (*B*, *B′*). These brain sections were used for counting and the boxed regions represent the area of the barrel cortex where the countings were performed. Statistical significance was assessed, using Student's *t*-test (*P* value < 0.05). Error bars represent the standard error of mean. Scale bars: 150 μm.

From the embryonic analysis we observed that the majority of MGE-derived interneurons, missing both Rac1 and Rac3, did not manage to migrate and populate the cortex on time (Fig. 1). Instead, they were found accumulated in the ventral telencephalon even postnatally (data not shown). Given the great reduction in migrating interneurons during embryogenesis, we analyzed the distribution (Supplementary Fig. 2) and numbers of interneuron subpopulations with specific markers at P5, after they reached their final position in the cortex (Fig. 3). For comparison we used equivalent cryosections from the double-mutant cortex and from the control cortex at different anterior–posterior levels (Fig. 3*B,B′*). A striking 80% of YFP^+^ cells were absent in the double-mutant barrel cortex compared with the control barrel cortex (Fig. 3*A,A′,C*). Comparisons between control and Rac3 mutant for YFP^+^ cells in the P15 cortex showed no statistically significant differences (data not shown). By using specific interneuron markers (Lhx6, GABA, PV) and quantifying the double-positive cells we demonstrated that in all cases, only 20% of double-labeled interneurons were found in the cortex of double-mutant mice (Fig. 3*D,E,F* and Supplementary Fig. 2). The same 80% reduction was observed after quantification of cells positive for Sst mRNA (Fig. 3*G*). The percentages of YFP^+^ cells that co-express GABA, Lhx6, and PV over the total number of YFP^+^ cells were not significantly different between control and mutants deficient in Rac1/Rac3 (Fig. 3*I–K*), indicating that the ability of the double-mutant precursors to differentiate into the various mature interneuron subtypes is not affected despite the great reduction in absolute numbers.

The caudal ganglionic eminence (CGE) gives rise to CR, vasoactive intestinal peptide (VIP) and neuropeptide Y (NPY) interneurons. We analyzed the number and distribution of CGE-derived interneuron subtypes (Fig. 3*H*, Supplementary Fig. 3) to see if the loss of MGE-derived interneurons could induce alterations in CGE-derived interneurons. Our data demonstrate that the number of CGE-derived cells is not altered in the cortex of the double mutants.

Given the great loss of interneurons, we decided to look for further abnormalities in the cortices of double mutants and examined the overall morphology along the AP and DV axes as well as the cortical layers in postnatal brains. No gross anatomical defects were observed apart from the smaller size of their brains in the AP axis (Supplementary Fig. 4 and data not shown). To check whether cortical layers are formed normally and whether pyramidal neurons are affected by the severe reduction of interneurons, we performed *in situ* hybridization using specific layer markers such as Rorβ, ER81, and Cux2. We also used the Rac3 probe, which is highly expressed in layer IV/V. We observed that all layers were present at the correct place, while the cortex width in the double-mutant animals was not significantly smaller compared with control animals (Supplementary Fig. 4).

Taken together, our results show that the postnatal cortex of double-mutant animals exhibits a great and specific reduction in the number of cortical MGE-derived interneurons. 80% of cortical interneurons are not found in the cortex, but the relative numbers of each subpopulation in the remaining 20% is not affected. Overall, our data point to a migration rather than a differentiation defect in MGE-derived interneurons.

### The Significant Reduction of MGE-derived Cells is in Part due to a Delay of Cell Cycle Exit

The absence of Rac1 causes a delay in cell cycle exit of MGE cells (Vidaki et al. 2012). Since Rac3 is also expressed in the MGE, we aimed to look into its possible additional contribution to the cell cycle exit. Control and double-mutant progenitor cells were pulse-labeled with BrdU at E12.5 and E14.5, respectively and a chase of 24 h was performed. BrdU was used to reveal the cells in S phase and the Ki67 marker for cycling cells (Supplementary Fig. 5*A*). The fraction of BrdU^+^ cells that did not express Ki67 in the MGE of double-mutant embryos (Supplementary Fig. 5*B*,*C*) was decreased, indicating that fewer cells were exiting the cell cycle in the absence of Rac1/Rac3 proteins, compared with controls. However, there was no difference in the cell cycle exit index between the Rac1 conditional mutants and the double mutants, suggesting that this effect is likely due to the absence of Rac1 alone (compare Supplementary Fig. 5 of this report and Fig. 5 of Vidaki et al. 2012). This result excludes the possibility that the more severe reduction of the MGE-derived cells in the cortex of the double-mutant mice is due to a further decrease in cell cycle exit.

Another possibility that could underlie the phenotype observed is cell death due to apoptosis. However, immunostaining of coronal sections from E13.5 and E15.5 embryos for activated caspase 3 did not reveal any differences between controls and double mutants (Supplementary Fig. 6). These results indicate that cell death may not be the primary cause of the major reduction of cortical interneurons in the absence of both Rac1 and Rac3. In agreement with these data, from midembryonic stages on, we observed an accumulation of YFP^+^ cells in the basal forebrain, close to their place of birth. However, it is possible that cells die gradually over a protracted period of time, making the assessment of cell death difficult due to low signal detection.

### MGE-derived Interneurons Missing Rac1 and Rac3 Have Morphological Defects

**Figure 4.**
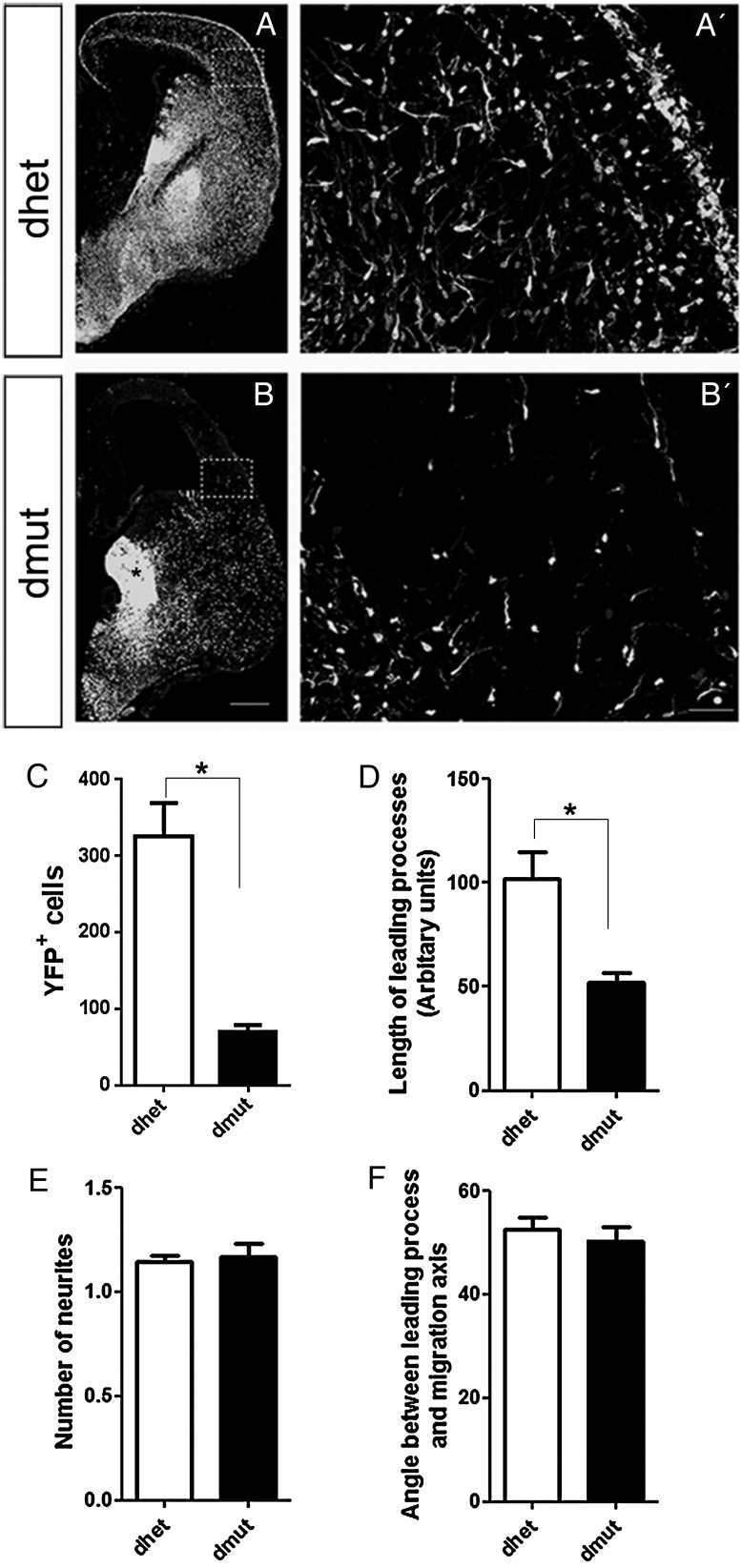
In vivo the migrating Rac1/Rac3-deficient cells have shorter leading process length compare with the control cells. Coronal sections from E15.5 control and Rac1/Rac3 double-mutant embryos were stained with an anti-GFP antibody (*A*, *B*). *A′* and *B′* high magnification of the boxed areas in *A*, *B* used for quantifications. The number of YFP^+^ cells (*C*), the length of the leading processes (*D*), the number of neurites (*E*), and the angle between the leading processes and migration axis (*F*), were determined and statistical significance was assessed, using Student's *t*-test (*P* value < 0.05, *n* = 20). Error bars represent the standard error of mean. Scale bars: *A*, *B* 300 μm; *A′* and *B′* 50 μm.

Rho-GTPases control cell cytoskeleton and morphology in several contexts. We monitored the morphology of YFP^+^ migrating cells in vivo at E15.5 (Fig. 4*A,B*) in the double mutants by YFP immunostaining. Very few YFP^+^ Rac1/Rac3-deficient cells have entered the dorsal telencephalon (Fig. 4*C*), with shorter leading processes (Fig. 4*D*) without any change in the number of processes per cell (Fig. 4*E*) and the angle the leading process forms with the presumptive migration axis when compared with controls (Fig. 4*F*). The absence of Rac1 alone revealed shorter neurites and defective lamellipodia formation when compared with control cells in the ventrally aggregated YFP^+^ population grown in vitro (Fig. 7 of Vidaki et al. 2012).

**Figure 5.**
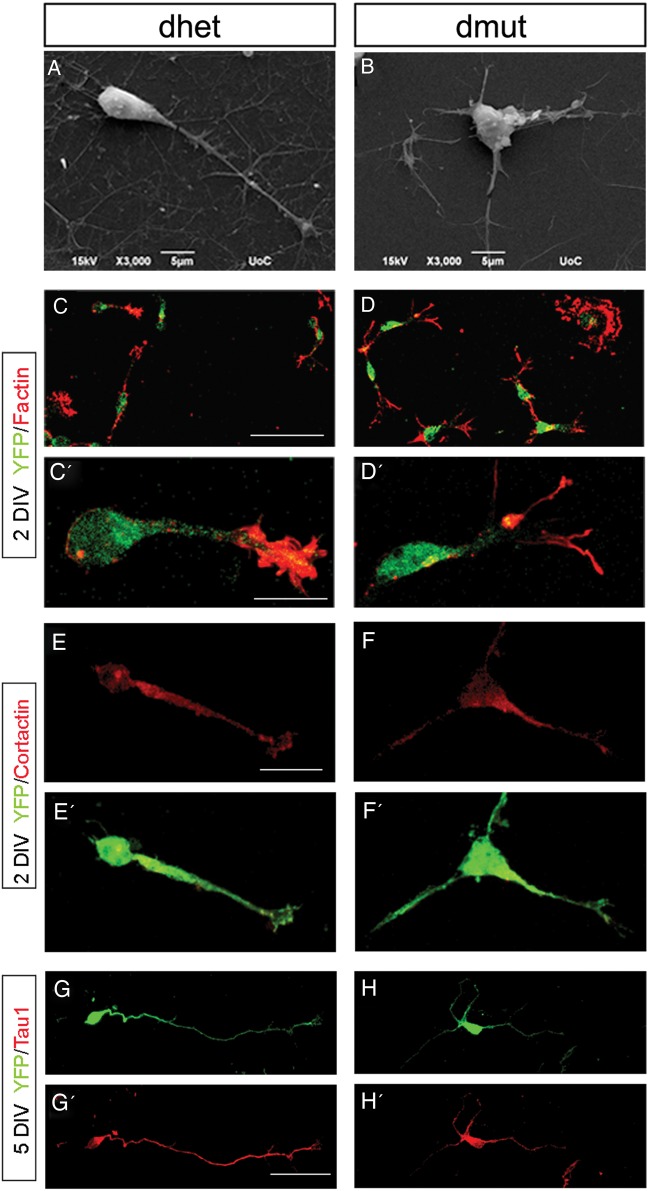
Morphological defects of Rac1/Rac3-deficient MGE-derived cells. The morphology of control (*A*; *C*, *C′*) and double-mutant (*B*; *D*, *D′*) interneurons was visualized by scanning electron microscopy (SEM) and YFP/Phalloidin immunohistochemistry in MGE explant cultures. These assays revealed the splitting of the leading process and the increase in neurite numbers in YFP^+^ double-mutant cells. Cortactin and YFP immunostaining of control (*E*, *E′*) and double-mutant (*F*, *F′*) interneurons revealed the absence of lamellipodia in the YFP^+^ double-mutant cells. MGE-derived cells cultured for 5 DIV from control (*G*, *G′*) and double-mutant (*H*, *H′*) E13.5 embryos were immunostained with anti-GFP and anti-Tau1 antibodies (*G–H′*). Tau1 immunostaining (*G′*, *H′*) shows signal in all neurites of double-mutant cells in contrast to the single neurite of control cells. Scale bars: *A* and *B*: 5 μm; *C* and *D*: 50 μm; *C′* and *D′*: 10 μm; *E*, *F*, *E′*, and *F′*: 20 μm; *G*, *H*, *G′*, and *H′*: 75 μm.

**Figure 6.**
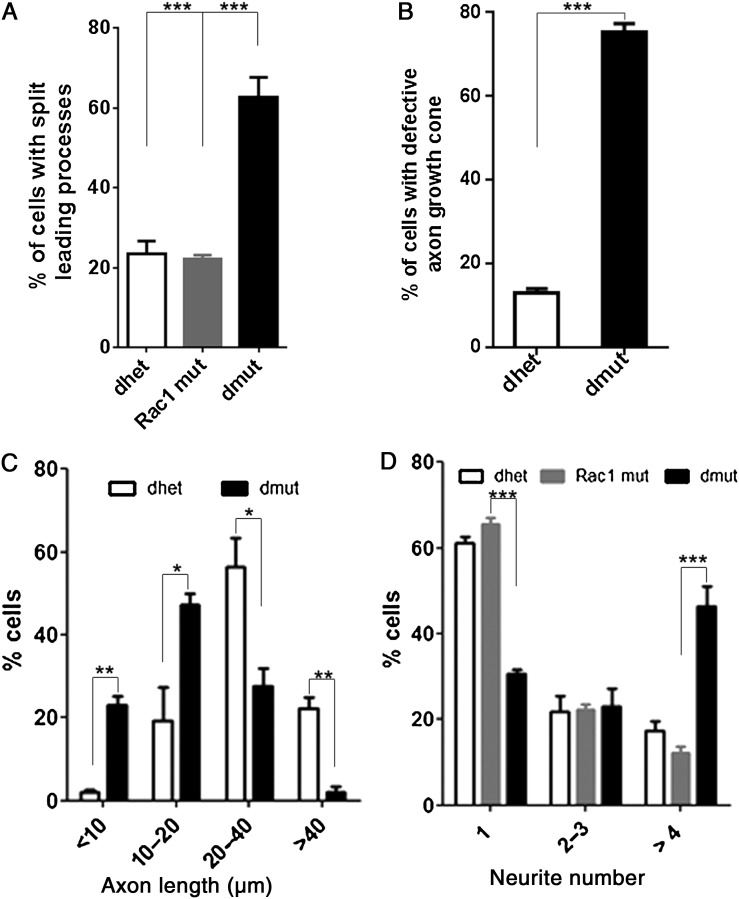
Rac1/Rac3-deficient interneurons have split-end leading processes and increased number of neurites. The percentages of the cells with split-end leading processes (*A*), presence of axon growth cone (*B*), leading process length, (*C*) and number of neurites (*D*) were calculated and statistical significance was assessed, using Student's *t*-test (*P* value ≪0.05). Error bars represent the standard error of mean.

To further study the morphological defects of Rac1/Rac3-deficient interneurons and their intrinsic or extrinsic nature, we grew ventrally aggregated YFP^+^ cells from the MGE on collagen-coated coverslips. We processed these cultures either for scanning electron microscopy (SEM) analysis or for immunostaining for various cytoskeletal markers. High magnification analysis of these cultures using SEM strongly indicated the presence of morphological defects in the absence of both Rac proteins (Fig. 5*A,B*). These morphological defects included an increased number of neurites per cell, splitting of the leading processes and absence of an obvious axonal growth cone. Immunostaining for YFP and Phalloidin to visualize the actin cytoskeleton in similar cultures (Fig. 5*C–D′*), revealed extensive splitting of the leading processes of double mutant cells after 2 DIV, a phenotype that is not frequently observed in control or single Rac1 conditional mutants (Fig. 5*C′,D′* and Fig. 6*A*).

We further investigated individual components pertaining to the cytoskeleton of the double-mutant cells compared with controls. By performing immunolabelling for cortactin we observed that lamellipodia formation was significantly impaired to the point that very few cortactin-positive structures were present in the double-mutant cells (Fig. 5*E,F,E′,F′* and Fig. 6*B*).

Other than the split-end aspect of the leading process and the defective growth cones, the majority of double-mutant cells displayed an increased number of neurites compared with control or Rac1 mutant cells, the majority of which possessed only one. About half of YFP^+^ cells from double-mutant MGEs presented more than 4 neurites, while this percentage was significantly reduced in the control YFP-positive MGE-derived cells and the Rac1 mutant cells (Fig. 6*D*).

In order to investigate further the nature of the multiple neurites observed in double-mutant cells, we used the axonal marker Tau1 to immunolabel cells isolated from the MGE and cultured for 5 DIV (Fig. 5*G–H′*). The Tau1 signal was evident in all neurites of double-mutant cells when compared with controls, where the majority of control cells had only one Tau1-positive neurite. This could indicate that the double-mutant cells present additional defects than Rac1 mutant cells such as disarrangement of leading processes and impaired polarity.

Overall, our analysis reveals that ablation of both Rac1 and Rac3 from MGE-derived interneurons results in severe morphological alterations, such as impaired neurite growth, increased number of processes per neuron, decreased neurite length and growth cone abnormalities. Our findings support the idea that Rac proteins are the master coordinators of these processes in MGE-derived interneurons.

### Microtubule Stability is Affected When Rac1 and Rac3 are Ablated

**Figure 7.**
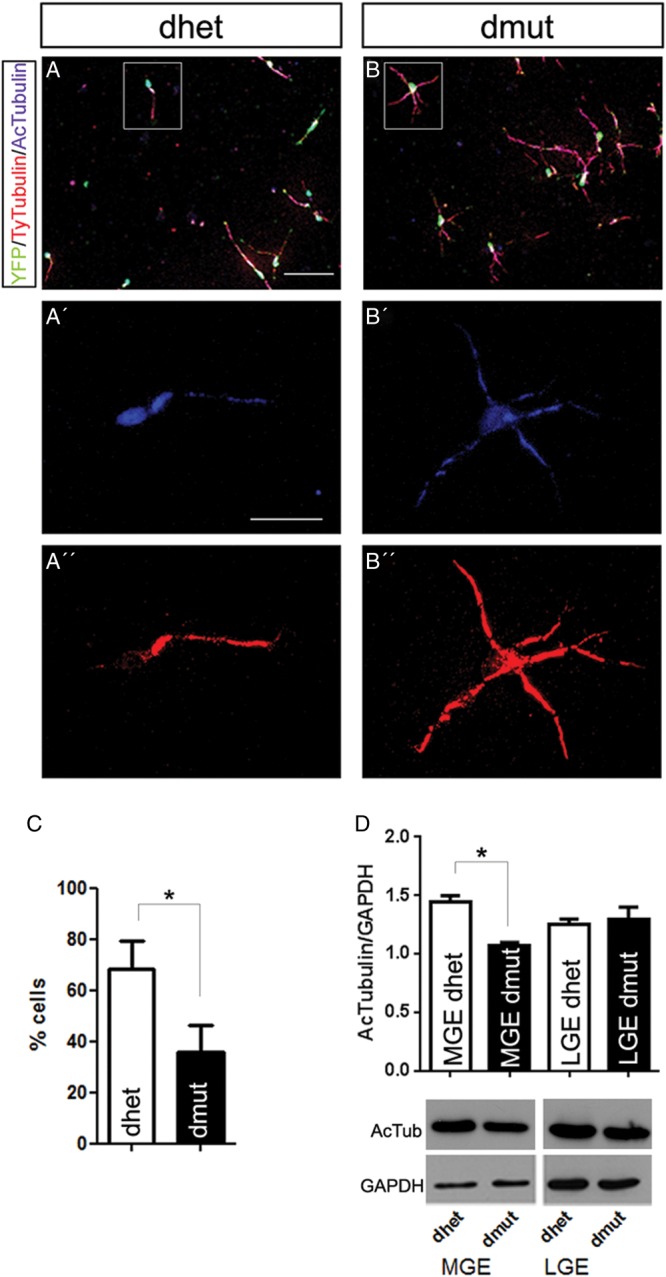
The distribution and the amount of Ac-Tubulin are different in the Rac1/Rac3-deficient MGE-derived cells. Cells cultured on collagen-coated coverslips were stained with antibodies against: YFP, Ac-Tubulin, and Ty-Tubulin (*A*, *B*). *A′*, *B′*, *A″* and *B″* represent high magnification of the boxed areas in *A*, *B*. (*C*) Quantification of Ac-Tub^+^ neurites at AIS showed a decrease in double-mutant cells. The Ac-Tubulin amount was determined after western blot analysis using MGE-derived cells (*D*) and statistical significance was assessed, using Student's *t*-test (*P* value < 0.05). Error bars represent the standard error of mean. Scale bars *A* and *B*: 75 μm; *A′*, *B′*, *A″*, and *B″*: 20 μm.

Post-translational modifications such as tubulin acetylation are correlated with stable microtubules, enriched in the proximal part of axons and at the tip of the leading processes (Janke and Kneussel 2010). We looked for the stability of microtubules in our cultures and found that the signal for Ac-Tubulin was enriched at the axon initial segment and cell body in the cells isolated from the MGE of control animals, but this was not the case in the Rac1/Rac3-deficient cells (Fig. 7*A′–B′,C*). Moreover western blot analysis showed that the amount of Ac-Tubulin was reduced in protein extracts from double-mutant MGE-derived cells (Fig. 7*D*). These data are consistent with the hypothesis that in the absence of Rac1/Rac3 proteins the stability of microtubules is affected and this, along with the actin defects reported above (Fig. 5), could cause the morphological defects of leading processes.

**Figure 8.**
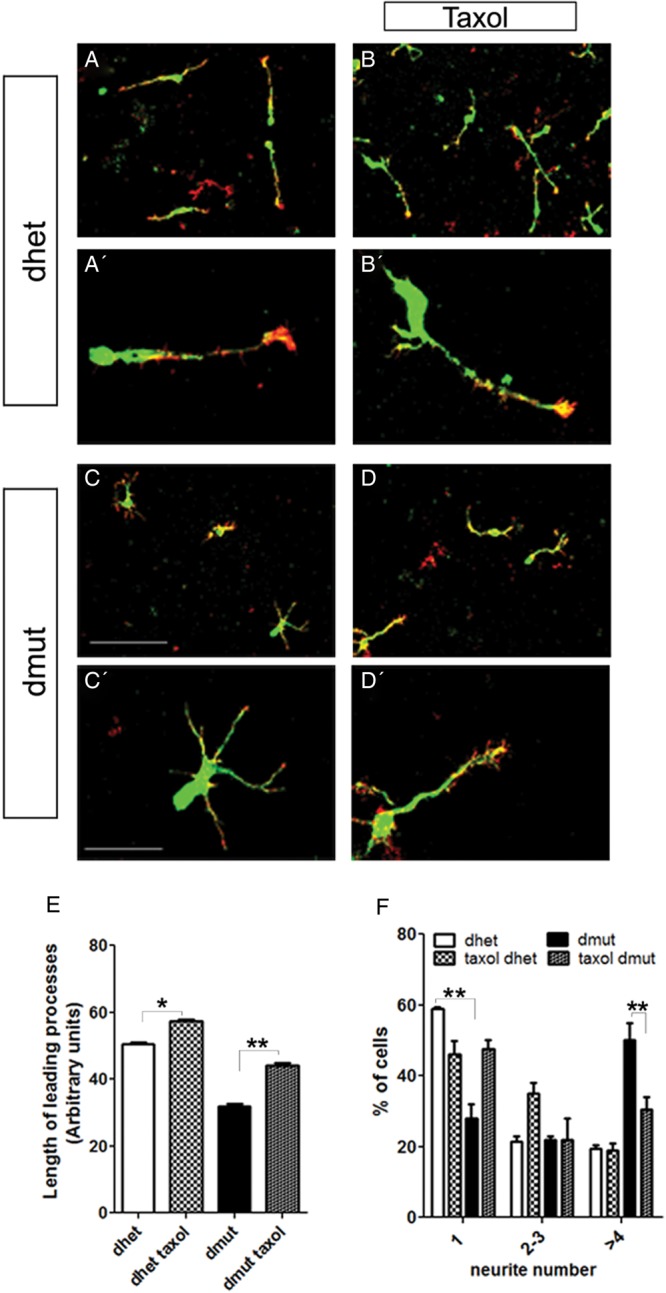
The abnormal morphology of Rac1/Rac3-deficient interneurons is changed by treatment with taxol. MGE-derived cells were cultured on collagen-coated coverslips for 24 h, followed by a period of 48 h culture in the presence of taxol. Subsequently the cells were processed for immunohistochemistry against Phalloidin-Alexa593 conjugated and GFP antibodies (*A–D′*). The length and the number of leading processes were measured (*E*, *F*) and statistical significance was assessed, using Student's *t-*test (*P* value ≪0.05). After the treatment with taxol, the length of the leading process was increased. In addition, the taxol-treated double-mutant cells presented fewer neurites per cell then the untreated cells (*F*). Scale bars *A*, *B*, *C*, and *D*: 75 μm; *A′*, *B*, *C′*, and *D′*: 25 μm.

The reduction in the amount of Ac-Tubulin in the double-mutant MGE-derived cells could be an indication that the amount of stable microtubules is also affected. We treated MGE-derived cells from control and double mutants in culture with taxol, which is known to stabilize microtubules by reducing their dynamics (Etienne-Manneville 2010). We observed a partial rescue after taxol treatment assessed by the length and the number of leading processes in the double-mutant cultures (Fig. 8). Leading process length was increased and the number of processes was reduced in the presence of taxol in cells where both Rac1 and Rac3 proteins were deleted (Fig. 8*E,F*).

These findings indicate that Rac proteins are essential for interneuron migration by regulating actin–microtubule dynamics in developing MGE-derived interneurons.

## Discussion

In this manuscript we address the role of Rac1 and Rac3 in the early development of cortical GABAergic interneurons. We show that the combined absence of Rac1 and Rac3 in MGE-derived cortical interneurons leads to a severe reduction of this neuronal population in the postnatal cortex in comparison to control and Rac1 single mutants. PV and Sst-expressing interneurons are 80% fewer in the cortex of Rac1/Rac3 double-mutant mice, compared with control animals. During embryonic development, interneurons missing both Rac-proteins are severely delayed in entering the neocortex while most of them remain aggregated ventrally in the basal telencephalon and are found there till early postnatal ages. These aggregated cells have abnormal morphology when cultured in vitro. In particular, most of these cells exhibit an increased number of neurites per cell, splitting of the leading processes and absence of an obvious growth cone. In short, the defects observed due to the combined absence of Rac1/Rac3 are additive but also distinct when compared with the absence of Rac1 alone which impairs lamellipodia formation (Vidaki et al. 2012). We propose that the defects arise in MGE progenitors (due to the absence of Rac1 as shown in Vidaki et al. 2012) but also in postmitotic interneurons since Rac1/Rac3-deficient cells aggregated in the SVZ exhibit gross morphological defects due to affected cytoskeletal dynamics (Fig. 5). Although we show that Rac1 and Rac3 proteins can partially compensate for each other in their functions, our results clearly demonstrate the distinct roles of those proteins during CNS development. We propose a role for Rac1 and Rac3 together as master regulators of the actin–microtubule cytoskeleton of cortical interneurons.

### Rac3 has a Compensatory Effect on GABAergic MGE-derived Interneurons When Rac1 is Ablated

Recent findings showed that Rac1 and Rac3 are synergizing to control aspects of the development of hippocampal and cortical interneurons via the use of Synapsin-Cre, a line that expresses Cre at E14.5 on, relatively late in cortical interneuron development (Vaghi et al. 2012). Our study is in good agreement with the Vaghi paper regarding the reduction in the number of postnatal interneurons, although the 2 studies differ in terms of the reduction extent and the subpopulations affected, presumably due to the Synapsin-Cre line that is expressed in differentiated neurons. The data using the Nkx2.1-Cre line, expressing in the MGE already at E11.5 have been shown to affect only the MGE-derived cells and shows a severe reduction ∼80% only in PV^+^ and Sst^+^ interneurons, the subpopulations originating from the MGE according to published information (Fogarty et al. 2007). This report focuses on embryonic events of cortical interneuron development affected by Rac1 and Rac3 and also examines the cellular/molecular correlates of the absence of Rac1/Rac3.

Rac1 is known to coordinate the migration of different cell types including neurons (see also next section). A migration impairment of cerebellar granule neurons occurs when Rac1 is ablated (Tahirovic et al. 2010). These neurons never express other Rac proteins. Although diverse functions have been attributed to Rac1, to a large extent depending on the cell type involved, the function of the neuronal specific Rac, Rac3, is less well-studied.

Our in vivo analysis shows a significant delay of MGE-derived YFP^+^ cells migrating to the cortex in the double-mutant when compared with the single Rac1-deficient interneurons (Vidaki et al. 2012). The migration of YFP^+^ cells in the absence of Rac3 alone was equivalent to the control animals and this is in line with the fact that no major migratory defects are found in Rac3 mutants (Corbetta et al. 2005). Our in vitro analysis that corroborates the in vivo findings, indicates that the migration defect is cell autonomous.

### Rac1 and Rac3 Regulate Leading Process Formation via the Actin and Microtubule Cytoskeleton

Rho-GTPases play important roles in crucial events during the development of many cell types of different organisms. They are shown to act redundantly in axon pathfinding and migration from *Caenorhabditis elegans* to mammalian neurons (Lundquist et al. 2001; Hakeda-Suzuki et al. 2002; Luo 2002; Ng et al. 2002; Lundquist 2003; Gonzalez-Billault et al. 2012). A significant degree of conservation among species is also observed in Rac interactors (Demarco et al. 2012; Doi et al. 2013; Law et al. 2013).

Rho-GTPases also participate in the dynamic assembly, disassembly, and reorganization of the actin and microtubule cytoskeleton (Hall and Lalli 2010). Microtubules together with actin filaments are the principal components of the cytoskeleton and are required to define cell morphology (Conde and Caceres 2009; Stiess and Bradke 2011). In addition, the cross-talk between the microtubule and actin cytoskeleton contributes to neuronal morphogenesis (Gonzalez-Billault et al. 2012). A multitude of studies have focused on the role of Rac1 in these processes in different cell types. Particularly in neuronal cells, Rac1 has been shown to be important in axon growth and lamellipodia formation. In vivo and in vitro migration of cerebellar granule neurons is Rac1-dependent and is controlled by the WAVE complex, Arp2/3 and actin remodeling in a cell autonomous manner (Tahirovic et al. 2010). At the same time, few studies address the role of Rac3 itself. One such study finds that overexpression of Rac3 in retinal ganglion cells induces the formation and increases the branching of new neurites (Albertinazzi et al. 1998).

Rac1-deficient interneurons exhibit growth cones with altered morphology and severely underdeveloped lamellipodia (Vidaki et al. 2012). These findings indicate that the polymerization and depolymerization of F-actin which are the processes regulating growth cone motility and axon outgrowth (Dent et al. 2011) are altered in the absence of Rac1. The consequence of these defects, in addition to the cell cycle exit deregulation, could be responsible for the migration delay of cortical interneurons in vivo and in vitro*.* By deleting both Rac proteins in cortical interneurons, we observed additional defects such as the reduction of leading process length in vivo and the appearance of extensive splitting of the leading processes in vitro*.* These observations indicate that Rac1 and Rac3 together may coordinate events important in cytoskeleton dynamics, required for proper migration of cortical GABAergic interneurons.

### Stabilization of Microtubules Improves Neuronal Growth and Polarity

Microtubules are the principal players involved in axonal extension (Conde and Caceres 2009; Dent et al. 2011). Their feature of dynamic stability enables growth of the axon shaft. On the other hand, microtubules are more stable than the actin cytoskeleton (Neukirchen and Bradke 2011). When nocodazole, a drug that inhibits microtubule dynamics, is used on migrating cortical interneurons, their morphology is altered (Baudoin et al. 2008). These alterations are reminiscent of the ones we observe in the double-mutant cells in culture, such as multipolar appearance with thin and labile processes.

When grown in vitro*,* half of the double-mutant cells isolated from the MGE present >4 Tau1^+^ neurites in comparison to the single Tau^+^ neurite of control cells. The expression of Tau is known to coincide with the establishment of an axon in neurons and it is a well-characterized axonal marker, not expressed by other types of neuronal processes (Barnes and Polleux 2009). We hypothesize that Rac1 and Rac3 have a role in the polarization of MGE-derived cells. It is well known that many regulators including Rho-GTPases, PI3K, Par-complex are involved in the polarization process and regulate the actin cytoskeleton (Conde and Caceres 2009). It is unclear at the moment, which is the principal regulator of polarity but one hypothesis places Rac1 upstream of Cdc42 (Fuchs et al. 2009). However, it is likely that the exact mechanism of the regulation of polarity may be cell type specific (Etienne-Manneville and Hall 2003; Garvalov et al. 2007).

Microtubule stability is responsible for neuronal polarization (Witte and Bradke 2008). A link has been proposed between Rac proteins and microtubule stabilization involving DOCK7 upstream and stathmin downstream of Rac, thus affecting neuronal polarity and axon formation (Watabe-Uchida et al. 2006). Acetylation, a post-transcriptional modification of tubulin, characterizes the stable long-lived microtubules (Piperno et al. 1987; Robson and Burgoyne 1989; Janke and Kneussel 2010). The axons of polarized hippocampal neurons contain increased amounts of Ac-Tubulin. Moreover axons and minor neurites respond differently after treatment with taxol and have a different distribution of Ac-tubulin (Witte et al. 2008). The distribution of Ac-tubulin is altered in double-mutant neurons compared with control cells and is also significantly reduced when Rac1/Rac3 are deleted in the MGE. Our data indicate that when both Rac proteins are absent, the amount of stable microtubules is reduced and this could be the underlying cause for the splitting of their leading processes.

The stabilization feature of taxol on microtubules is well known although its exact mechanism of action is not fully understood. Taxol stabilizes microtubules by reducing their dynamics. Moreover, taxol changes the tubulin conformation leading to impairment of microtubule depolymerization (Xiao et al. 2006). It has been demonstrated that axonal microtubules show increased stability; when a caged form of taxol is used to stabilize one neurite, it was sufficient to transform it into an axon (Witte et al. 2008). In line with these findings, we observe an increase of the leading process length after treatment of our cultures with taxol in the absence of Rac1/Rac3 proteins. Moreover, the number of processes per cell is decreased after treatment with taxol in double-mutant cells. This indicates that the stabilization of microtubules could improve the defect of leading process growth and polarity. Our working hypothesis, corroborated by our in vitro experiments, is that microtubule dynamics is affected when both Rac1 and Rac3 are missing but not when only Rac1 is deleted.

These data support our hypothesis that Rac1 and Rac3 proteins together play an important role in the coordination of actin and microtubule dynamics which is necessary for polarity and axon elongation, processes that are prerequisite for the proper migration of GABAergic interneurons.

## Supplementary Material

Supplementary can be found at: http://www.cercor.oxfordjournals.org/.

## Funding

S.T. was funded by the EU Marie Curie |PERG7-GA-2010-268292, ESPA 2007-2013 Postdoctoral Fellowships LS7-629, ESPA 2007-13 GSRT 11 ROM22-4-ET29, State Scholarships Foundation (IKY) Fellowship, Z.K. by the ERC-01 of the GSRT, Greek Ministry of National Education and Religious Affairs and K.K. from the graduate program of Molecular Biology-Biomedicine of the University of Crete during 2011–2012. Furthermore we acknowledge support from the University of Crete (Special Accounts) and intramural funds of the IMBB (to D.K.) as well as the Medical Research Council grant-in-aid U117537087 (to V.P.). Funding to pay for the Open Access publication charges for this article was provided by the National Institute for Medical Research, MRC.

## Supplementary Material

Supplementary Data
