# Supplementary material for: Rac-GTPases Regulate Microtubule Stability and Axon Growth of Cortical GABAergic Interneurons
Source: Cereb Cortex. 2014 Mar 13;25(9):2370–82. doi: 10.1093/cercor/bhu037 (PMC4537417; doi:10.1093/cercor/bhu037)
Supplement: Supplementary Data [file supp_25_9_2370__index.html]

Rac-GTPases Regulate Microtubule Stability and Axon Growth of Cortical GABAergic Interneurons — Rac-GTPases Regulate Microtubule Stability and Axon Growth of Cortical GABAergic Interneurons — Rac-GTPases Regulate Microtubule Stability and Axon Growth of Cortical GABAergic Interneurons — Supplementary Data 

# Rac-GTPases Regulate Microtubule Stability and Axon Growth of Cortical GABAergic Interneurons

## Supplementary Data

Supplementary Data

**Files in this Data Supplement:**

- Supplementary Data - Doc file
- Supplementary Figure 1 - tif file
- Supplementary Figure 2 - tif file
- Supplementary Figure 3 - tif file
- Supplementary Figure 4 - tif file
- Supplementary Figure 5 - tif file
- Supplementary Figure 6 - tif file
