# Supplementary material for: Rac-GTPases Regulate Microtubule Stability and Axon Growth of Cortical GABAergic Interneurons
Source: Cereb Cortex. 2014 Mar 13;25(9):2370–82. doi: 10.1093/cercor/bhu037 (PMC4537417; doi:10.1093/cercor/bhu037)
Supplement: Supplementary Data [file supp_bhu037_bhu037supp.doc]

**Supplementary Figure 1. Analysis of Rac3 expression.**

In situ hybridization for Rac3 mRNA at E13.5 using coronal sections from the forebrain of developing wild type and Rac3 mutant embryos (A, B), E16.5 (C) using wild type and at P5 using Rac1^+/fl^;Rac3^+/-^;Nkx2.1^+/Cre^ (D). (E) Total RNA was isolated from E13.5 MGE of wild-type (+/+) and knockout (Rac3^-/-^, -/-) mice, reverse transcribed and then used for PCR amplification with specific primers for Rac3 and GAPDH as internal control. The PCR amplification product was 580bp for Rac3 and 101bp for GAPDH. nc: negative control, M: λ/HindIII ladder. Scale bars A, B: 50μm, C, D: 100 μm.

**Supplementary Figure 2. MGE-derived cortical interneurons are severely reduced in Rac1/Rac3 deficient postnatal barrel cortex.**

Coronal sections from P5 and P15 brains were immunostained for specific markers of GABAergic interneurons: Lhx6 (A, A`), GABA (B, B`), Sst (C, C`), PV (D, D`) together with YFP and after these cells were counted in the barrel cortex. (A-D) include representative areas of the barrel cortex from controls and (A`-D`) from Rac1/Rac3 mutants. The inserted images in C, C` showed the in situ hybridization with Sst probe from the barrel cortex sections that were used for counting the total number of Sst^+^ Scale bars: 75 μm.

**Supplementary Figure 3. Number of CGE-derived cells in Rac1/Rac3 deficient postnatal barrel cortex.**

Coronal sections from P15 and P5 brains were analyzed by IHC analysis using antibodies against: YFP, CR (A, A`) and by ISH using the NPY probe (B, B`).

Scale bars: 75 μm.

**Supplementary Figure 4. Cortical lamination is not affected by the deletion of Rac1 and Rac3.**

Coronal sections from P5 brains were analyzed by Nissl staining (A, F) and in situ hybridization using markers for different cortical layers: Rac3 (B, G), Ror beta for layer IV (C, H), Cux2 for upper layers (D, I) and ER81 for layer V (E, J).

(**A`-J`**) represent high magnification of the barrel cortex area from (A-J). Scale bars: 100 μm.

**Supplementary Figure 5. Rac1/Rac3 deletion leads to reduced exit of GABAergic interneurons from the cell cycle.**

(A), double immunohistochemistry for BrdU (blue) and Ki67 (red) was performed on E13.5 coronal sections of the developing forebrain of control and double mutant embryos, obtained 24 hours after BrdU injection. (B, C), Cell cycle exit was assessed, by calculating the percentage of BrdU^+^;Ki67^-^ cells to the total number of BrdU^+^ cells in the SVZ of the MGE in four sets of embryos. This percentage was decreased in the MGE of Rac1/Rac3-deficient embryos, compared to their control littermates.

Scale bars: 37,5 μm.

**Supplementary Figure 6. Rac1/Rac3 deficiency does not induce apoptotic cell death of MGE derived interneurons.** Immunohistochemistry for active Caspase 3 on coronal sections of E13,5 (A, B) forebrains indicated the same number of apoptotic cells at the MGE level between control (A) and double mutant (B) embryos. Scale bars: 75 μm.
