# Supplementary figures and images for: Rac-GTPases Regulate Microtubule Stability and Axon Growth of Cortical GABAergic Interneurons
Source: Cereb Cortex. 2014 Mar 13;25(9):2370–82. doi: 10.1093/cercor/bhu037 (PMC4537417; doi:10.1093/cercor/bhu037)

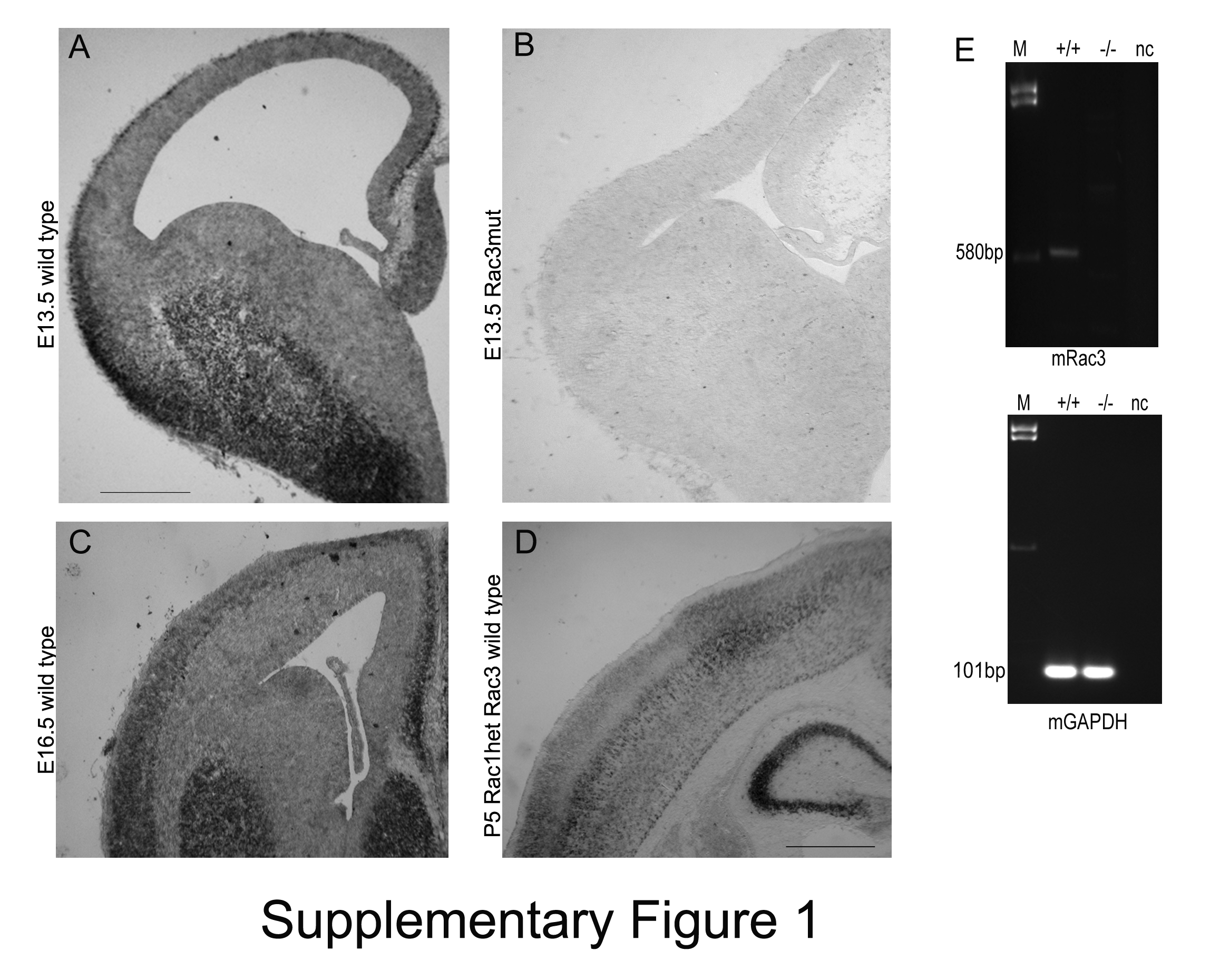

Supplement: Supplementary Data [file supp_bhu037_bhu037supp_fig1.tif]

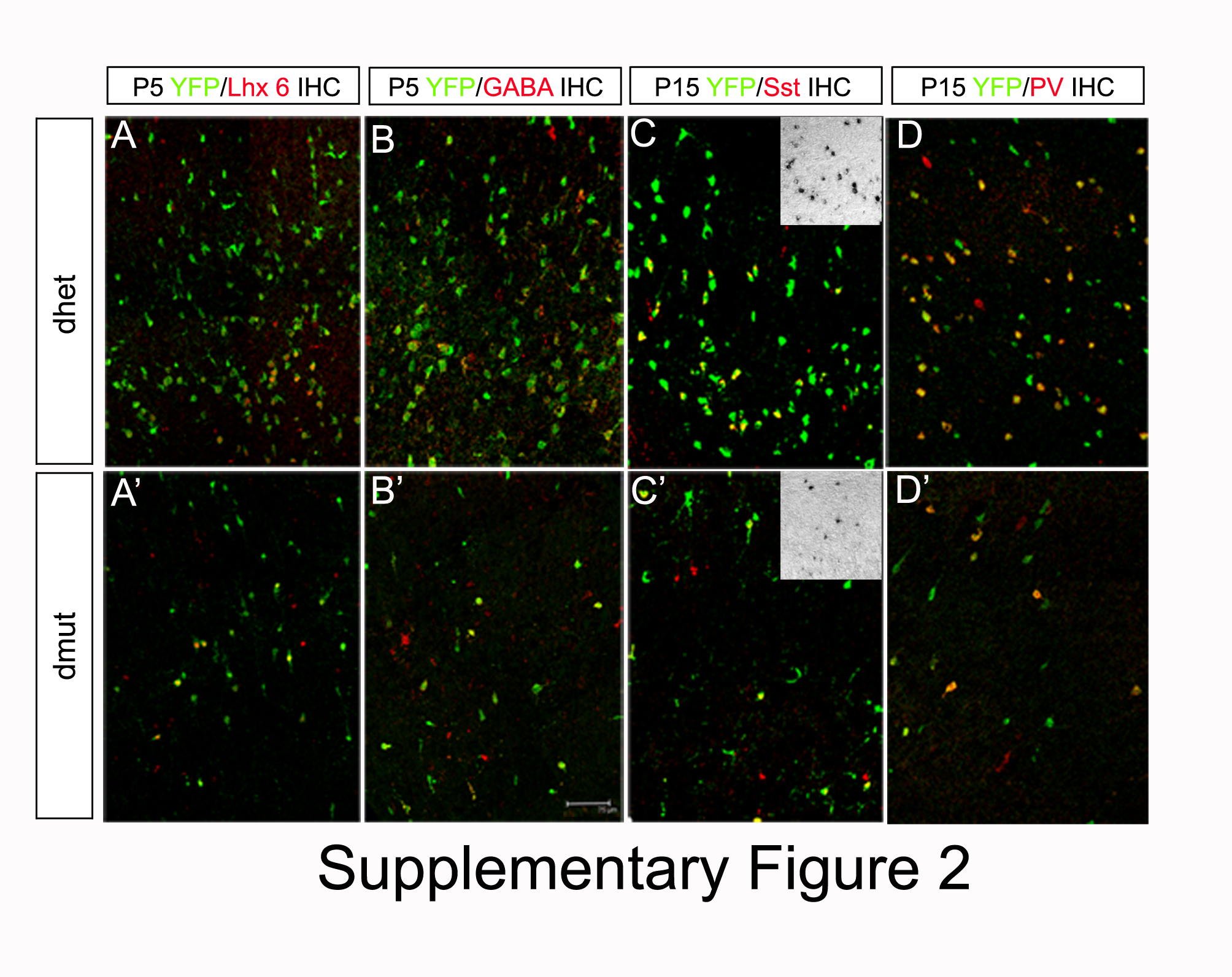

Supplement: Supplementary Data [file supp_bhu037_bhu037supp_fig2.tif]

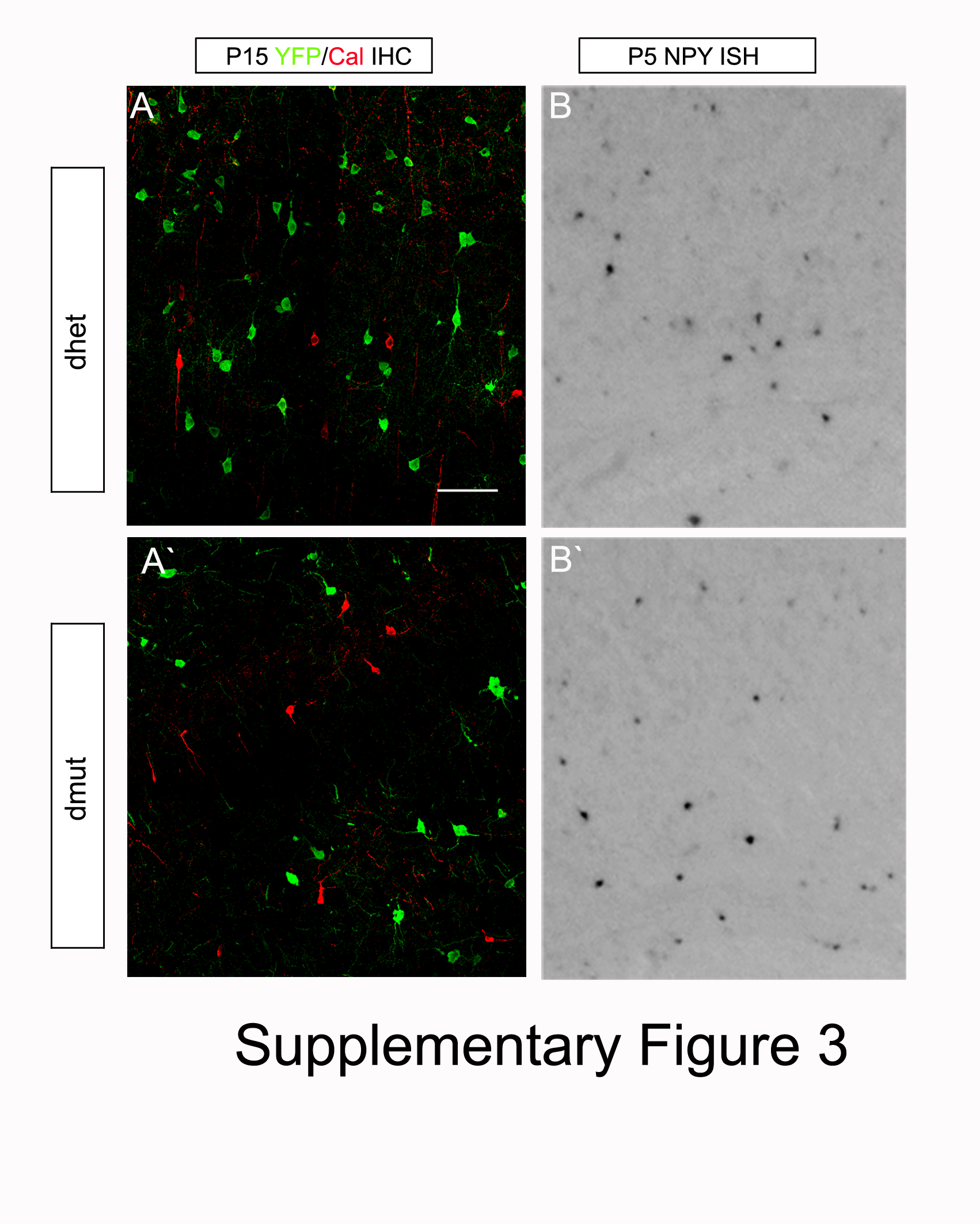

Supplement: Supplementary Data [file supp_bhu037_bhu037supp_fig3.tif]

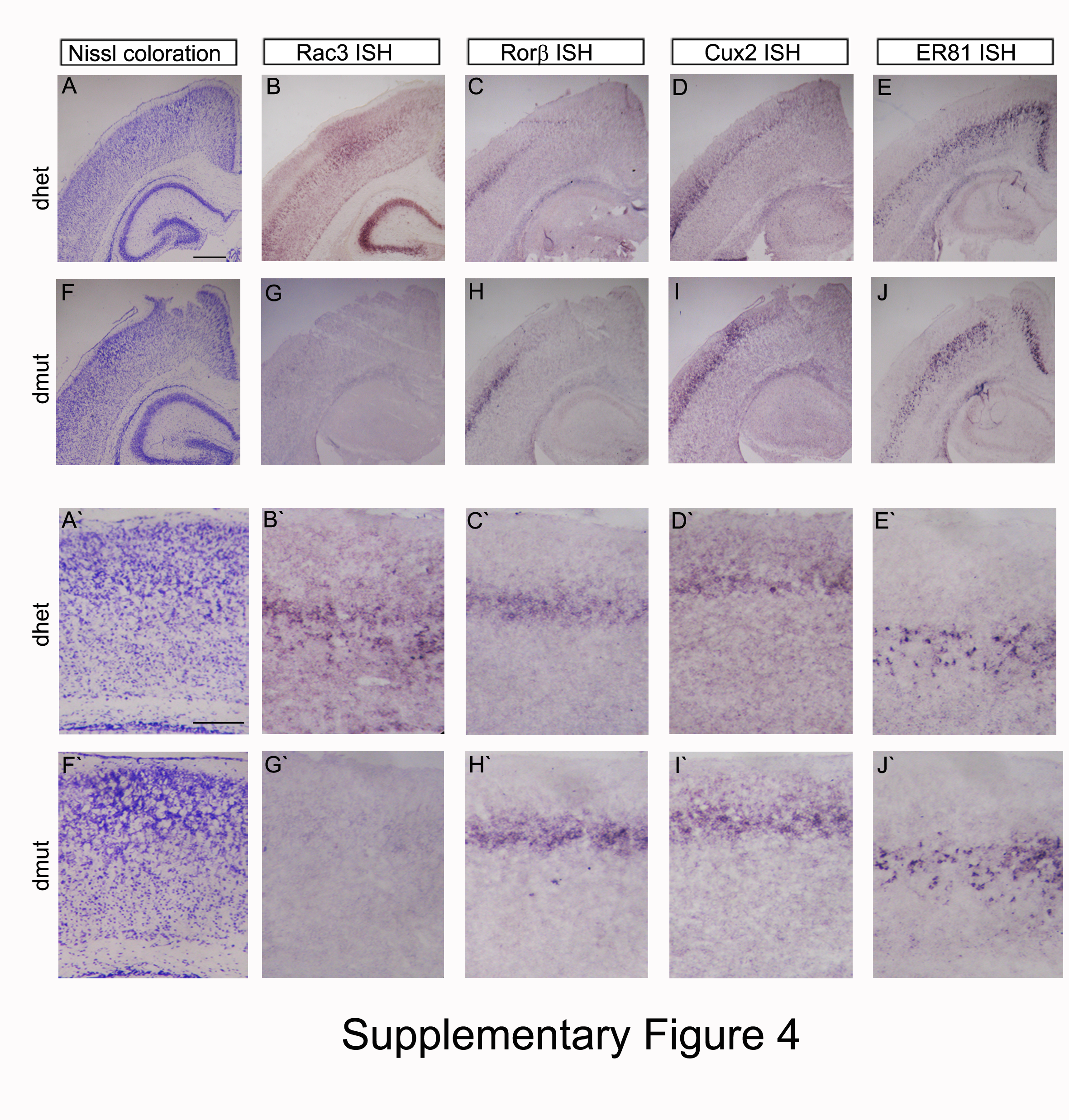

Supplement: Supplementary Data [file supp_bhu037_bhu037supp_fig4.tif]

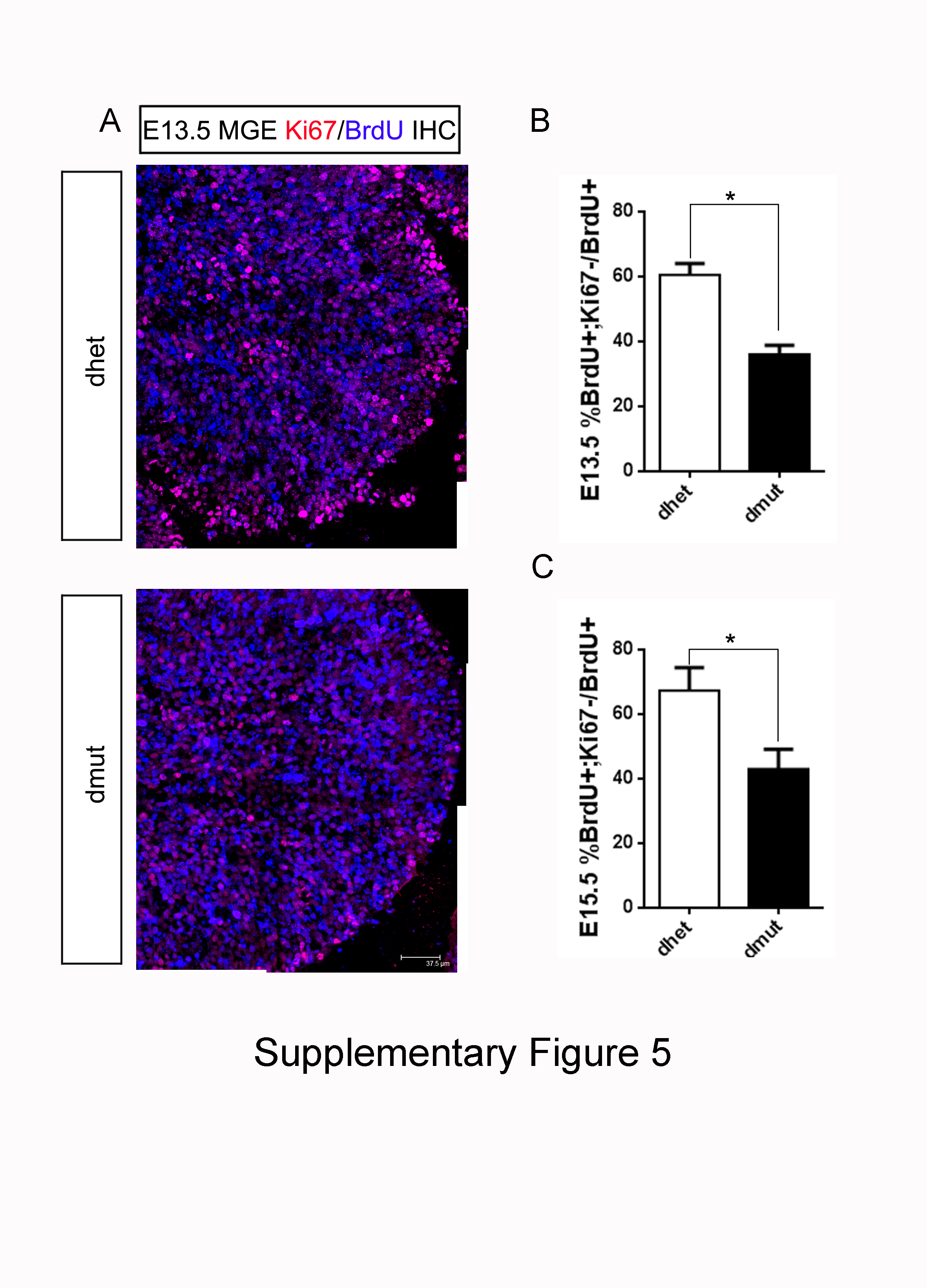

Supplement: Supplementary Data [file supp_bhu037_bhu037supp_fig5.tif]

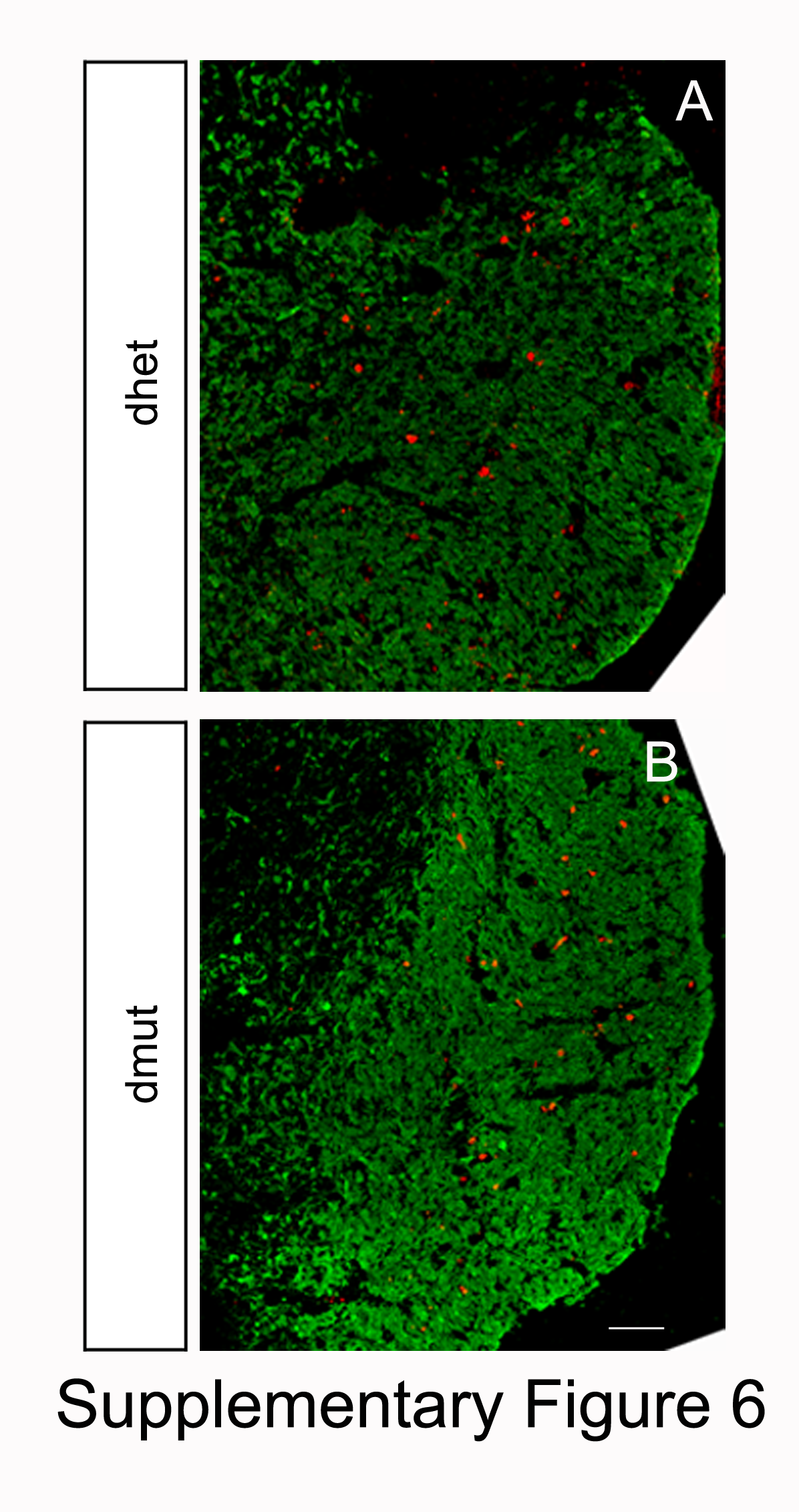

Supplement: Supplementary Data [file supp_bhu037_bhu037supp_fig6.tif]
